# Cinnamon Ameliorates Experimental Allergic Encephalomyelitis in Mice via Regulatory T Cells: Implications for Multiple Sclerosis Therapy

**DOI:** 10.1371/journal.pone.0116566

**Published:** 2015-01-08

**Authors:** Susanta Mondal, Kalipada Pahan

**Affiliations:** Department of Neurological Sciences, Rush University Medical Center, Chicago, IL, United States of America; Washington University, UNITED STATES

## Abstract

Upregulation and/or maintenance of regulatory T cells (Tregs) during an autoimmune insult may have therapeutic efficacy in autoimmune diseases. Although several immunomodulatory drugs and molecules are available, most present significant side effects over long-term use. Cinnamon is a commonly used natural spice and flavoring material used for centuries throughout the world. Here, we have explored a novel use of cinnamon powder in protecting Tregs and treating the disease process of experimental allergic encephalomyelitis (EAE), an animal model of MS. Oral feeding of cinnamon (*Cinnamonum verum*) powder suppresses clinical symptoms of relapsing-remitting EAE in female PLP-TCR transgenic mice and adoptive transfer mouse model. Cinnamon also inhibited clinical symptoms of chronic EAE in male C57/BL6 mice. Dose-dependent study shows that cinnamon powder at a dose of 50 mg/kg body wt/d or higher significantly suppresses clinical symptoms of EAE in mice. Accordingly, oral administration of cinnamon also inhibited perivascular cuffing, maintained the integrity of blood-brain barrier and blood-spinal cord barrier, suppressed inflammation, normalized the expression of myelin genes, and blocked demyelination in the central nervous system of EAE mice. Interestingly, cinnamon treatment upregulated Tregs via reduction of nitric oxide production. Furthermore, we demonstrate that blocking of Tregs by neutralizing antibodies against CD25 abrogates cinnamon-mediated protection of EAE. Taken together, our results suggest that oral administration of cinnamon powder may be beneficial in MS patients and that no other existing anti-MS therapies could be so economical and trouble-free as this approach.

## Introduction

Regulatory T cells (Tregs) are regarded as the master regulator of immune responses because this cell type maintains the homeostasis between immune activation and immune suppression [1, 2]. Tregs suppress activation and proliferation of self-reactive T cells and thereby inhibit immune response of self-reactive T cells against self-antigens [1, 2]. Tregs are characterized by the presence of transcription factor forkhead box p3 (Foxp3) and therefore, Foxp3^+^ CD4^+^CD25^+^ T cells are considered as the most common phenotype of Tregs [1, 3]. Under normal physiological conditions, Tregs are able to suppress self-reactive T cells. However, during autoimmune pathogenesis, the immune system is dysregulated, resulting in a substantial decrease in the activity and the number of Tregs, and thereby leading to proliferation of self-reactive T cells and subsequent autoimmune attack.

It is increasingly becoming clear that Tregs play important roles in multiple sclerosis (MS) and its animal model experimental autoimmune encephalomyelitis (EAE). MS is associated with deficiency of Treg numbers and function [4, 5]. It has been shown that Tregs play a critical role in protection and recovery from EAE [6]. Although the exact mechanism of protection by Tregs is not clearly understood, it is suspected that Tregs exert protection by increasing the Th2 phenotype and decreasing the homing of autoreactive T cells [6]. Depletion of CD4^+^CD25^+^ cells inhibits natural recovery from EAE, whereas transfer of these cells to recipient mice reduces disease severity [7]. These observations imply that regulation of Tregs might play a decisive role in susceptibility to EAE. Recent studies suggest that the expression of Foxp3 and the numbers of peripheral CD4^+^CD25^+^ Foxp3^+^ T cells are significantly reduced in relapsing-remitting MS patients compared with those in control subjects [8]. Therefore, upregulation and/or maintenance of Tregs may be beneficial for MS.

Although there are some immunomodulatory compounds [9, 10], here we have tested a novel approach to achieve immunomodulation. Cinnamon is a commonly used natural spice and flavoring material used for centuries throughout the world. Here we delineate that cinnamon treatment increased the expression of Foxp3 and enriched Tregs in EAE mice via suppression of nitric oxide production. Accordingly, cinnamon treatment suppressed Th1 and Th17 responses and augmented Th2 response *in vivo* in EAE mice. Finally, cinnamon treatment was capable of ameliorating the disease process of relapsing-remitting EAE in two different models in female mice and chronic EAE in male mice. Furthermore, abrogation of the cinnamon-mediated protection from EAE by anti-CD25 antibody suggests that cinnamon protects against EAE via Tregs. These results suggest that cinnamon may be used to control autoimmune pathologies in MS via upregulation/maintenance of Tregs.

## Materials and Methods

Animal maintaining and experiments were in accordance with National Institute of Health guidelines and were approved by the Institutional Animal Care and Use committee (IACUC#11–005) of the Rush University of Medical Center, Chicago, IL. Animals exhibiting paralysis were kept on soft bed and fed and watered through animal feeding needles. However, if any mouse came to the moribund stage, it was decapitated after anesthesia with ketamine/xylazine injectables.

### Reagents

Bovine myelin basic protein (MBP), L-glutamine and β-mercaptoethanol were obtained from Invitrogen (Carlsbad, CA). Fetal bovine serum (FBS) and RPMI 1640 were from Mediatech (Washington, DC). Sodium benzoate (NaB), sodium formate (NaFO), solvent blue 38, cresyl violet acetate, and lithium carbonate were purchased from Sigma Aldrich (St. Louis, MO). Original Ceylon cinnamon (*Cinnamonum verum*) in ground form was obtained from Indus Organics (San Ramon, CA). Heat-killed *M. tuberculosis* (H37RA) was purchased from Difco Labs. Incomplete Freund’s adjuvant (IFA) was obtained from Calbiochem.

### Screening of PLP-TCR transgenic (Tg) mice

PLP_139–151_-specific 5B6 TCR Tg mice were obtained from Prof. Vijay Kuchroo (Harvard Medical School, Boston, MA). These mice were genotyped by flow cytometry. Briefly, a drop of blood was collected from tail bleed into 200 µl PBS in a 96 well plate. Samples were spun, RBCs were lyzed and cells were stained with Thy1.1, CD4 and Vβ6. When gated on CD4+ cells, the homozygotes were positive Thy1.1 and 90% or greater cells were positive for Vβ6.

### Induction of EAE

**Adoptively-transferred EAE.** It was performed as described previously by us [11, 12, 13, 14]. Briefly, 4–5 weeks old female SJL/J mice were purchased from Harlan Sprague-Dawley (Indianapolis, IN). Donor mice were immunized s.c. with 400 µg bovine MBP and 60 µg *M. tuberculosis* in IFA [11, 12, 13, 14]. Animals were killed 10–12 days postimmunization, and the draining lymph nodes were harvested and single cell suspensions were cultured in RPMI 1640 supplemented with 10% FBS, 50 µg/mL MBP, 50 µM 2-ME, 2 mM L-glutamine, 100 U/mL penicillin, and 100 µg/ml streptomycin. On day 4, cells were harvested and resuspended in HBSS. A total of 2 × 10^7^ viable cells in a volume of 200 µL were injected into the tail vein of naive mice. Pertussis toxin (150 ng/mouse; Sigma-Aldrich) was injected once via i.p. route on 0 day post-transfer (dpt) of cells. Animals were observed daily for clinical symptoms. Six mice were used in each group. Female mice (4–5 week old) were randomly selected for any group. Experimental animals were scored by a masked investigator, as follows: 0, no clinical disease; 0.5, piloerection; 1, tail weakness; 1.5, tail paralysis; 2, hind limb weakness; 3, hind limb paralysis; 3.5, forelimb weakness; 4, forelimb paralysis; 5, moribund or death.

A mouse was considered moribund when it showed any of the following criteria. Conditions for moribund were as follows: Prolonged inappetance; Evidence of muscle atrophy; Central nervous system disturbance (Head tilt, Seizures, Tremors, Circling, Spasticity, and Paresis); Chronic diarrhea or constipation; Rough coat and distended abdomen; Spreading area of alopecia caused by disease; Coughing, rales, wheezing and nasal discharge; Distinct jaundice and/or paleness (anemia); Markedly discolored urine, polyuria or anuria; Inability to remain upright; Frank bleeding from any orifice; Persistent self-induced trauma.

**Figure 1:**
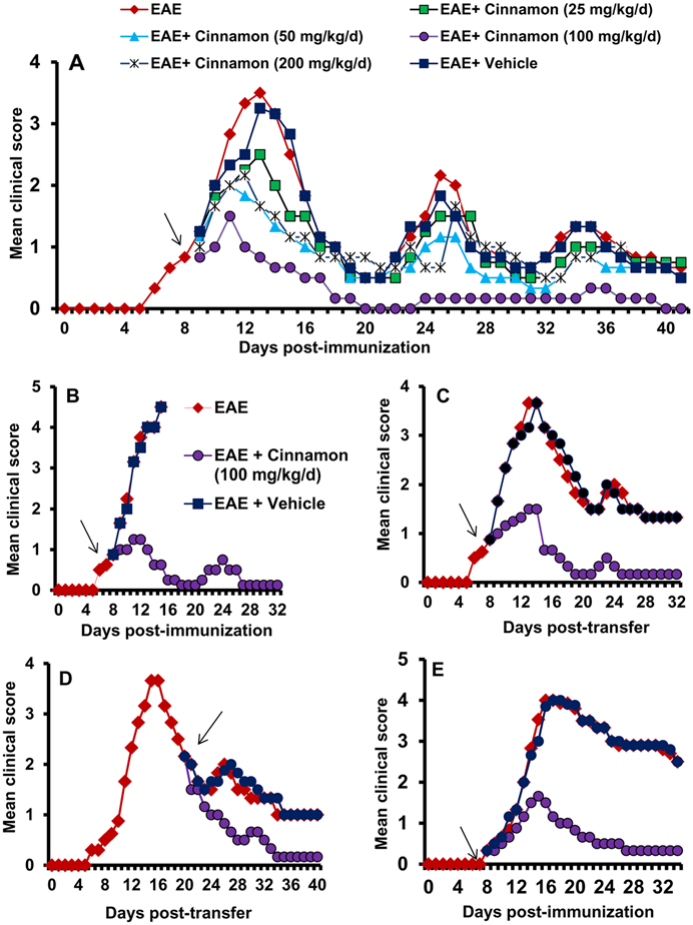
Oral administration of ground cinnamon suppresses clinical symptoms of EAE in female PLP-TCR transgenic (Tg) mice, adoptive transfer model in female SJL/J mice and chronic model in male C57/BL6 mice. A) PLP-TCR Tg mice were immunized with 10 μg of PLP139–151, and from 8 days post- immunization (dpi) mice were treated with different doses of ground cinnamon or vehicle (0.1% methylcellulose) via gavage. Mice (*n* = 6 in each group) were scored daily until 41 dpi. B) PLP-TCR Tg mice were immunized with 25 μg of PLP139–151, and from 8 dpi mice were treated with ground cinnamon (100 mg/kg body wt/d) via gavage. Mice (*n* = 6 in each group) were scored daily until 32 dpi. C) EAE was induced in female SJL/J recipient mice by adoptive transfer of MBP-primed T cells. From 8 dpt, mice were treated with either cinnamon (100 mg/kg body wt/d) or vehicle via gavage. Mice (*n* = 6 in each group) were scored daily until 32 dpt. D) In this adoptive transfer model, mice were also treated with either cinnamon or vehicle from 20 dpt. Mice (*n* = 6 in each group) were scored daily until 40 dpt. E) C57BL/6 mice were immunized with 100 μg of MOG35–55, and from 8 dpi, mice were treated with either cinnamon or vehicle. Mice (*n* = 6 in each group) were scored daily until 34 dpi.

**Relapsing EAE in 5B6 PLP-TCR Tg mice.** Female Tg mice (4–5 weeks old) were immunized with 10 or 25 µg of PLP139–151 in *M. tuberculosis* in IFA as described above. Mice also received pertussis toxin (150 ng/mouse) once on 0 day post-immunization (dpi). In the EAE group (Fig. 1B), where female PLP-TCR transgenic mice were immunized with 25 μg PLP139–151, two mice died without humane intervention on 17 days post-immunization (dpi) and four moribund mice were decapitated after anesthesia. However, according to the disease scale, all six mice in this group received a score of 5.

**Chronic EAE.** C57BL/6 mice were immunized with 100 μg of MOG35–55 as described above. Mice also received two doses of pertussis toxin (150 ng/mouse) on 0 and 2 dpi.

### Cinnamon treatment

Cinnamon (*Cinnamonum verum*) powder was mixed in 0.5% methylcellulose (MC) and EAE mice were gavaged 100 µL cinnamon-mixed MC powder once daily using gavage needle as described [15, 16]. Therefore, control EAE mice received only MC as vehicle.

### Histological microscopy

On 14 dpi (first chronic phase), five mice from each of the following groups (control, EAE, EAE+cinnamon, and EAE+vehicle) were anesthetized. After perfusion with PBS (pH 7.4) and then with 4% (w/v) paraformaldehyde solution in PBS, cerebellum and whole spinal cord was dissected out from each mouse. The tissues were further fixed and then divided into halves: one-half was used for histological staining whereas the other half was used for myelin staining as described earlier [11, 12, 13, 14]. For histological analysis, routine histology was performed to obtain perivascular cuffing and morphological details of CNS tissues of EAE mice. Paraformaldehyde-fixed tissues were embedded in paraffin, and serial sections (4 µm) were cut. Sections were stained with conventional H&E staining method. Digital images were collected under bright-field setting using an x40 objective. Slides were assessed in a blinded fashion by three examiners for inflammation in different anatomical compartments (meninges and parenchyma). Inflammation was scored using the following scale as described: for meninges and parenchyma: 0, no infiltrating cells; 1, few infiltrating cells; 2, numerous infiltrating cells; and 3, widespread infiltration. For vessels: 0, no cuffed vessel; 1, one or two cuffed vessels per section; 2, three to five cuffed vessels per section and 3, more than five cuffed vessels per section. At least six serial sections of each spinal cord from each of five mice per group were scored and statistically analyzed by ANOVA.

### Staining for myelin

Sections were stained with Luxol fast blue for myelin as described earlier [13, 14]. Slides were assessed in a blinded fashion for demyelination by three examiners using the following scale: 0, normal white matter; 1, rare foci; 2, a few areas of demyelination; and 3, large areas of demyelination. At least six serial sections of each spinal cord from each of five mice per group were scored and statistically analyzed by ANOVA.

### Semi-quantitative RT-PCR analysis

Total RNA was isolated from splenic T cells and spinal cord by using the RNeasy mini kit (Qiagen, Valencia, CA) and from spleen and cerebellum by using the Ultraspec-II RNA reagent (Biotecx laboratories, Inc, Houston, TX) following manufacturer’s protocol. To remove any contaminating genomic DNA, total RNA was digested with DNase. Semi-quantitative RT-PCR was carried out as described earlier [13, 14] using a RT-PCR kit from Clonetech (Mountain View, CA). Briefly, 1 µg of total RNA was reverse transcribed using oligo(dT)_12–18_ as primer and MMLV reverse transcriptase (Clontech) in a 20 µL reaction mixture. The resulting cDNA was appropriately-diluted, and diluted cDNA was amplified using Titanium Taq DNA polymerase and following primers. Amplified products were electrophoresed on a 1.8% agarose gels and visualized by ethidium bromide staining.

Foxp3: Sense, 5'-CAG CTG CCT ACA GTG CCC CTAG-3'
Antisense, 5'-CAT TTG CCA GCA GTG GGT AG-3’
CD25: Sense, 5'-AGC CAA GTA GGG TGT CTC TCA ACC-3'
Antisense, 5'-GCC CAG GAT ACA CAG TGA AGA ACG-3'
CD4: Sense, 5’- CCA ACA AGA GCT CAA GGA GAC CAC-3’
Antisense, 5’- CGT ACC CTC TTT CCT AGC AAA GGA-3’
CD62L: Sense, 5’- AGC CTC TTG CCA GCC AGG GT-3’
Antisense, 5’- CCA GCC CCG AGA ATG CGG TG-3’
CTLA4: Sense, 5’- GGT CCG GGT GAC TGT GCT GC-3’
Antisense, 5’- CCC GTT GCC CAT GCC CAC AA-3’
IFN-γ: Sense, 5’- GCTGTTACTGCCACGGCACA-3’
Antisense, 5’- GGACCACTCGGATGAGCTCA-3’
T-bet: Sense, 5’- GGAGCGGACCAACAGCATC-3’
Antisense, 5’- CCACGGTGAAGGACAGGAAT-3’
IL-10: Sense, 5’- GCACTGCTATGCTGCCTGCT-3’
Antisense, 5’- CCGATAAGGCTTGGCAACCC-3’
GATA3: Sense, 5’- TCTGGAGGAGGAACGCTAATGG-3’
Antisense, 5’- GAACTCTTCGCACACTTGGAGACTC-3’
IL-17: Sense, 5’- GCTGACCCCTAAGAAACCCC-3’
Antisense, 5’- GAAGCAGTTTGGGACCCCTT-3’
iNOS: Sense: 5’-CCCTTCCGAAGTTTCTGGCAGCAGC-3’
Antisense: 5’-GGCTGTCAGAGCCTCGTGGCTTTGG3’
IL-1β: Sense: 5’-CTCCATGAGCTTTGTACAAGG-3’
Antisense: 5’-TGCTGATGTACCAGTTGGGG-3’
MBP: Sense: 5’-TGGAGAGATTCACCGAGGAGA-3’
Antisense: 5’-TGAAGCTCGTCGGACTCTGAG-3’
CNPase: Sense: 5’-CTACCCTCCACGAGTGCAAGA-3’
Antisense: 5’-AGTCTAGTCGCCACGCTGTCT-3’
GAPDH: Sense: 5'-GGTGAAGGTCGGTGTGAACG3'
Antisense: 5'-TTGGCTCCACCCTTCAAGTG-3'

The relative expression of each gene with respect to GAPDH was measured after scanning the bands with a Fluor Chem 8800 Imaging System (Alpha Innotech, San Leandro, CA).

### Real-time PCR analysis

It was performed using the ABI-Prism7700 sequence detection system (Applied Biosystems, Foster City, CA) as described earlier [13, 14]. Briefly, reactions were performed in a 96-well optical reaction plates on cDNA equivalent to 50 ng DNase-digested RNA in a volume of 25 µL, containing 12.5 µL TaqMan Universal Master mix and optimized concentrations of FAM-labeled probe, forward and reverse primers following the manufacturer’s protocol. All primers and FAM-labeled probes for mouse genes and GAPDH were obtained from Applied Biosystems. The mRNA expressions of respective genes were normalized to the level of GAPDH mRNA. Data were processed by the ABI Sequence Detection System 1.6 software and analyzed by ANOVA.

### Assay for NO synthesis

Synthesis of NO was determined by assay of culture supernatant for nitrite, a stable reaction product of NO with molecular oxygen, using ‘Griess’ reagent as described earlier [17, 18, 19].

### Flow cytometry

Two-color flow cytometry was performed as described previously [17, 18]. Briefly, 1 × 10^6^ lymph node cells (LNC) or splenocytes suspended in flow staining buffer were incubated at 4°C with appropriately diluted FITC-labeled Ab to CD4 for 30 min, washed, and resuspended in fixation and permeabilization solution. Following incubation in dark for 30 min, cells were washed, blocked with test Fc block (anti-mouse CD16/32) in permeabilization buffer, and subsequently incubated with appropriately diluted PE-labeled Abs to T-bet, IFN-γ, GATA3, IL-4, IL-17, RORγT, or Foxp3 at 4°C in the dark. After incubation, the cell suspension was centrifuged, washed thrice, and resuspended in flow staining buffer. The cells then were analyzed through FACS (BD Biosciences, San Jose, CA). Cells were gated based on morphological characteristics. Apoptotic and necrotic cells were not accepted for FACS analysis.

### Assay of suppressive activity of Tregs

Because cells from tomato red transgenic (B6.129(Cg)-Gt(ROSA)26Sor^tm4(ACTB-tdTomato,-EGFP)Luo^/J) mice exhibit red color, we used these mice (Jackson Laboratories, Bar Harbor, ME) for clear visualization of the suppressive activity of Tregs. These mice were immunized with MOG (100 µg/mouse) suspended in IFA containing 60 µg *M. tuberculosis* and 12 d after immunization, splenocytes were isolated and re-primed with MOG (10 µg/ml) for 2 d. These MOG-primed tomato red T cells expressed Th1 and Th17 cytokines (data not shown). In a parallel experiment, B6.129 mice were also immunized with MOG and treated with cinnamon for 2d followed by purification of CD4+CD25+ Tregs. Then these cinnamon-induced Tregs were added to MOG-primed splenocytes isolated from tomato red transgenic mice at a ratio of 2:1 of tomato red T cell:cinnamon-induced Tregs and the suppressive activity of Tregs was monitored by the inhibitory effect on the IFN-γ expression by MOG-primed tomato red T cells. Therefore, after 24 h of incubation, CD3+ T cells were purified and immunostained for IFN-γ (green). IFN-γ-expressing red cells were counted and expressed as percent of total red cells.

## Results

### Cinnamon inhibits clinical symptoms and disease severity of EAE in female PLP-TCR transgenic mice

Earlier we have demonstrated that cinnamon metabolite sodium benzoate (NaB) inhibits the adoptive transfer of EAE in female SJL/J mice [13]. Chinese cinnamon (*Cinnamonum cassia*) and original Ceylon cinnamon (*Cinnamonum verum* or *Cinnamonum zylencum*) are two major types of cinnamon that are available in the US. Recently by mass spectrometric analysis, we have found that *Cinnamonum verum* is much more pure than *Cinnamonum cassia* [15]. Although cinnamaldehyde is present as the major peak in both *Cinnamonum cassia* and *Cinnamonum verum*, *Cinnamonum cassia* contains more styrene, benzene, 1,1’-(2-butene-1,4-diyl)bis-, benzene, 1,1’-(1,2-cyclobutanediyl)bis-, palmitic acid, stearic acid, 4-phenylbutyl chloride, and (2,3-diphenylcyclopropyl)methyl phenyl sulfoxide than *Cinnamonum verum* [15]. Furthermore, *Cinnamonum cassia*, but not *Cinnamonum verum*, contains small amount of toxic 1-benzopyran-2-one or coumarin [15]. We have also found that oral treatment of mice with ground *Cinnamonum verum* via gavage markedly increases the level of NaB in serum and brain [15], suggesting that cinnamon is metabolized into NaB and that cinnamon-derived NaB is capable of entering into the CNS. Therefore, here, we examined whether oral administration of *Cinnamonum verum* powder ameliorates the disease process of RR-EAE in female PLP-TCR transgenic (Tg) mice [20]. Although these PLP-TCR Tg mice exhibited clinical symptoms of EAE spontaneously, clinical scores were low. Only 40% female PLP-TCR Tg mice developed EAE with a mean clinical score of 1.42 + 0.49. However, as reported [20], immunization with low dose (10 μg/mouse) of PLP139–151 strongly induced clinical symptoms of EAE (Fig. 1A). EAE mice were treated with different doses of cinnamon powder from 8 days post immunization (dpi) when these mice exhibited a clinical score of 0.5 or higher. An additional group of mice was treated with vehicle (Fig. 1A). Since the relapsing-remitting type of EAE is associated with multiple chronic phase peaks following the acute phase peak, we continued our observations until 42 dpt. Even at a dose of 25 mg/kg body wt/d, cinnamon significantly inhibited clinical symptoms (Fig. 1A & Table-1) with no decrease in disease incidence considering a clinical symptom of 1 or higher as disease incidence. On the other hand, at a dose of 100 mg/kg body wt/d, a dramatic inhibition of clinical symptoms and a significant reduction in disease incidence were observed in acute as well as chronic phases of EAE (Fig. 1A & Table-1). Vehicle (0.1% methyl cellulose) remained unable to inhibit the clinical symptoms of EAE (Fig. 1A & Table-1), suggesting the specificity of the effect. However, at a dose of 200 mg/kg body wt/d, cinnamon was less potent than either 50 or 100 mg/kg body wt/d in suppressing clinical symptoms (Fig. 1A & Table 1), suggesting that at higher dose, it may be toxic for EAE mice. We induced severe disease by immunizing these PLP-TCR Tg mice with 25 μg of PLP 139–151/mouse, where all mice died within 16 dpi (Fig. 1B). Even in this instance, cinnamon at a dose of 100 mg/kg body wt/d markedly suppressed clinical symptoms of EAE and no mouse from the cinnamon-treated group died during the course of the study (Fig. 1B). These findings demonstrate that cinnamon is capable of inhibiting clinical symptoms and disease severity in acute as well as chronic phases of EAE in PLP-TCR Tg mice.

**Table 1:** Effect of cinnamon on clinical symptoms of EAE in PLP-TCR Tg mice.

**Treatment**	**Incidence**	**Mean peak clinical score**	**Suppression of EAE**	
			**Incidence**	**Score**
EAE	6/6	3.6		
EAE+Cinnamon (25 mg/kg/d)	6/6	2.5	0	30.5 b
EAE+Cinnamon (50 mg/kg/d)	5/6	1.83	16.67	49 a
EAE+Cinnamon (100 mg/kg/d)	4/6	1.33	33.33 b	63 a
EAE+Cinnamon (200 mg/kg/d)	6/6	2.16	0	40 a
EAE+Vehicle	6/6	3.5	0	2.7

EAE was induced in female PLP-TCR Tg mice by immunization with 10 µg of PLP139–151 in *M. tuberculosis* in IFA. Mice also received pertussis toxin (150 ng/mouse) once on 0 day post-immunization (dpi). From 8 dpi, mice were treated with different doses of cinnamon emulsified in vehicle (0.5% methylcellulose) orally. While a clinical score of 1 was considered as the incidence of EAE in mice, a clinical score of 0 was considered to be normal. *^a^* *p* < 0.001, *^b^* *p* < 0.05 vs EAE (control).

### Cinnamon inhibits the adoptive transfer of EAE in female SJL/J mice

RR-EAE is also induced in female SJL/J mice by adoptive transfer of MBP-primed T cells [11, 13, 14]. Therefore, next, we examined if cinnamon treatment was also capable of suppressing the progression of EAE in this adoptive transfer model. Mice were treated with cinnamon powder in two different groups. In the first group, mice were treated with cinnamon from 8 days post-transfer (dpt), the onset of acute phase. An inhibitory effect of cinnamon on the clinical symptoms was clearly observed within a few days of treatment (Fig. 1C). Greater inhibition was observed on subsequent days of treatment, which was maintained throughout the duration (32 dpt) of the experiment (Fig. 1C). On the other hand, vehicle had no such inhibitory effect (Fig. 1C). In the second group, cinnamon treatment began from the onset of relapsing phase (20 dpt) and was continued until 40 dpt. Here, too, cinnamon, but not vehicle, halted the disease progression (Fig. 1D). However, in contrast to the first instance, the inhibitory effect of cinnamon was manifested after 6 days of treatment (26 dpt). The EAE disease severity in the cinnamon-treated group was close to 0 (normal) in the latter part of treatment (Fig. 1D). These results clearly demonstrate that cinnamon can ameliorate the ongoing relapsing-remitting EAE when administered either early (at the onset of acute phase) or late (at the onset of relapsing disease).

### Cinnamon inhibits chronic EAE in male C57/BL6 mice

While female SJL/J mice are used to induce RR-EAE, chronic form of EAE is modeled in male C57/BL6 mice upon immunization with MOG35–55. Next, we examined the efficacy of cinnamon powder in suppressing the disease process of chronic EAE. Similar to its effect on RR-EAE in female PLP-TCR Tg mice and SJL/J mice, cinnamon treatment also strongly inhibited the clinical symptoms of EAE in this chronic model (Fig. 1E). Again, vehicle had no effect on chronic EAE (Fig. 1E), suggesting the specificity of the effect.

### Cinnamon treatment inhibits the generation of encephalitogenic T cells in donor mice

**Figure 2:**
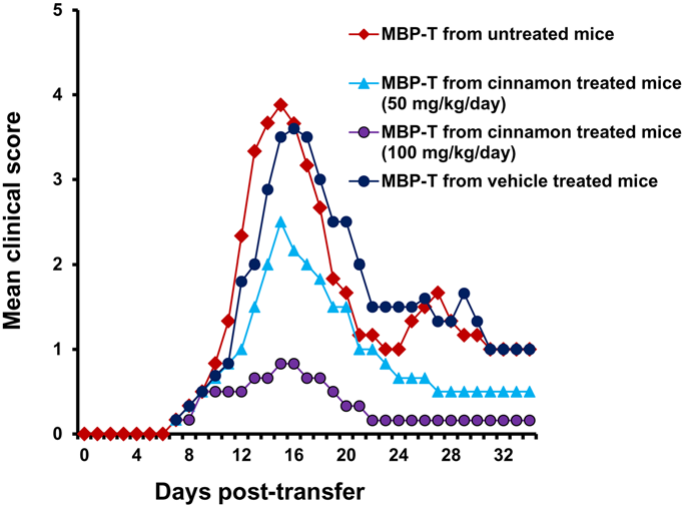
Oral administration of ground cinnamon inhibits the generation of encephalitogenic T cells *in vivo* in donor mice. Donor mice (4–6 week old female SJL/J) were immunized with MBP, IFA, and *M. tuberculosis.* From 2^nd^ day of immunization, mice were treated with either cinnamon (50 and 100 mg/kg body wt/d) or vehicle via gavage. On day 12 of immunization, mice were sacrificed, and total LNC were further primed with MBP (50 μg/ml) for 4 days. A total of 2 × 10^7^ viable MBP-primed T cells was adoptively transferred to naïve recipient mice. Six mice were used in each group. Mice were examined for clinical symptoms every day until 34 dpt.

As T cells isolated from MBP-immunized donor mice are encephalitogenic, and adoptive transfer of these T cells induces EAE in recipient mice, we investigated whether treatment of donor mice with cinnamon was capable of inhibiting the production of encephalitogenic T cells. In order to test this, donor mice were treated with different doses of cinnamon orally from the day of MBP immunization. T cells isolated from cinnamon-treated and untreated MBP-immunized donor mice were then adoptively transferred to recipient mice. Our results showed that mice receiving MBP-primed T cells from cinnamon-treated donor mice exhibited significantly reduced clinical symptoms and disease severity compared to mice receiving MBP-primed T cells from either untreated donor mice or vehicle-treated donor mice (Fig. 2). These results suggest that cinnamon treatment inhibits the generation of encephalitogenic T cells *in vivo* in donor mice.

### Cinnamon treatment preserves the integrity of blood-brain barrier (BBB) and blood-spinal cord barrier (BSB) in PLP-TCR transgenic mice

**Figure 3:**
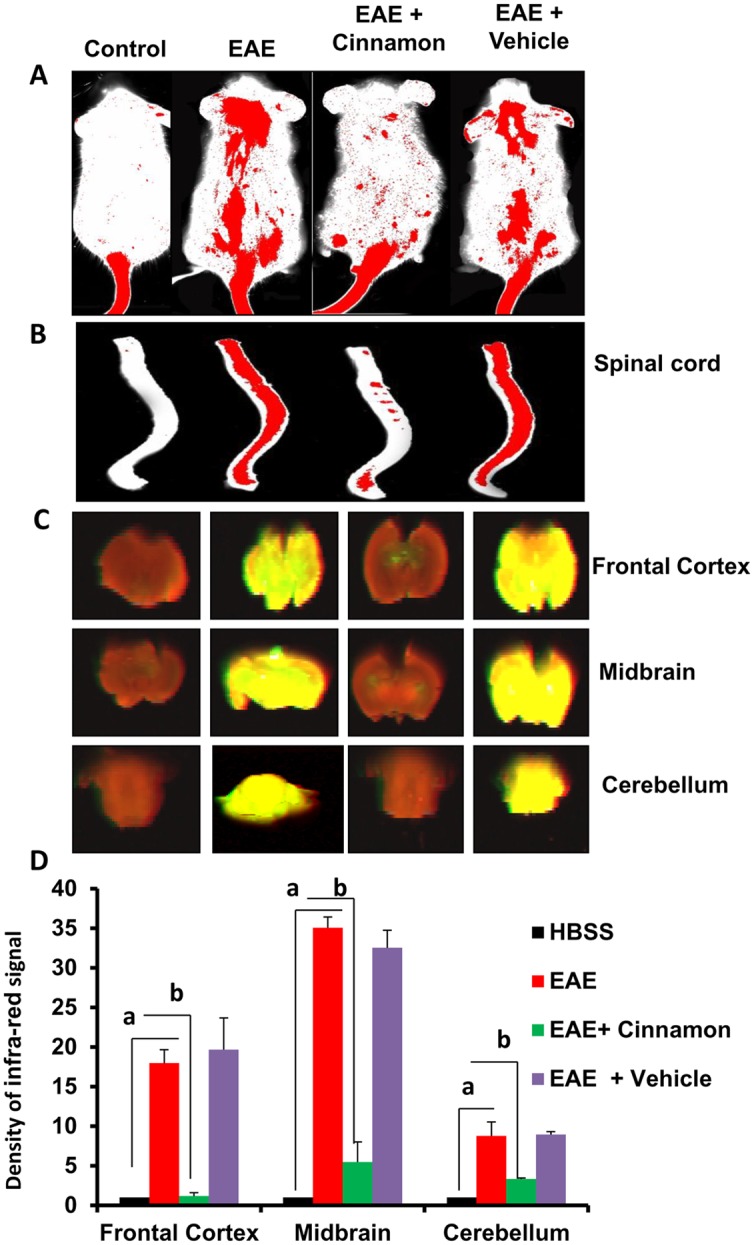
Effect of oral administration of cinnamon on the integrity of blood-brain barrier (BBB) and blood-spinal cord barrier (BSB) in EAE in PLP-TCR Tg mice. Control, EAE (14 dpi), and either cinnamon- or vehicle-treated EAE mice (14 dpi receiving cinnamon/vehicle from 8 dpi) (*n* = 5 in each group) received 200 μl of 20 μM Alexa Fluor 680-SE-NIR dye (Invitrogen) via the tail vain on 16 dpt (acute phase). After 4 h, mice were scanned in an Odyssey (ODY-0854; Licor) infrared scanner at the 700- and 800-nm channels (A). Mice were perfused with 4% paraformaldehyde. Spinal cord (*B*) and different parts of the brain (*C*) were scanned in an Odyssey infrared scanner. The red background came from an 800-nm filter, whereas the green signal was from Alexa Fluor 680 dye at the 700-nm channel. The density of the Alexa Fluor 680 signal in different parts of the brain (D) was quantified with the help of Quantity One, version 4.6.2 software, using the volume contour tool analysis module. Data are expressed as the mean ± SEM of five different mice; *^a^* *p*<0.001 vs normal (HBSS); ^b^p<0.001 vs EAE.

BBB and BSB are membranic structures that act primarily to protect the brain and the spinal cord respectively from chemicals in the blood, while still allowing some essential molecules to enter. It is known that during active MS and EAE, BBB and BSB break down in a section of the brain and spinal cord respectively due to widespread inflammation thereby allowing different blood molecules and toxins enter into the CNS. We investigated if cinnamon treatment modulated the integrity of BBB and BSB. We injected an infrared dye (Alexa-680) via tail-vein and 2 h after the injection, live mice were scanned in an Odyssey infra-red scanner. As evidenced from Fig. 3A (first lane), infra-red signals were not visible on areas over the brain and the spinal cord in control HBSS-injected mice. On the other hand, in EAE mice, infra-red signals were detected on areas over the brain and the spinal cord (Fig. 3A; second column), suggesting possible breakdown of BBB and BSB. However, cinnamon treatment strongly inhibited the entry of infra-red dye into the CNS of EAE mice (Fig. 3A; compare lane 3 with lane 2). In contrast, vehicle treatment did not influence the entry of infra-red dye into the CNS of EAE mice as evidenced by the aligning of infra-red signals over spinal cord and brain (Fig. 3A; compare lane 4 with lane 2), suggesting the specificity of the effect.

To confirm these results further, mice were sacrificed, and the spinal cord and different parts of the brain (frontal cortex, midbrain and cerebellum) were scanned for infra-red signals in Odyssey infra-red scanner. Consistent to live mice results, we did not notice much infra-red signal in the spinal cord and different parts of the brain in control HBSS-treated mice (Fig. 3B-D; lane 1) but significant amount of infra-red dye was visible in CNS tissues of EAE mice (Fig. 3B-D; lane 2). Again, treatment of EAE mice by cinnamon markedly attenuated the entry of infra-red dye into the spinal cord and different parts of the brain (Fig. 3B-D; compare lane 3 with lane 2). Taken together, these results suggest that cinnamon treatment preserves the integrity of BBB and BSB in EAE mice.

### Cinnamon inhibits infiltration of mononuclear cells, inflammation and demyelination in the spinal cord of EAE

**Figure 4:**
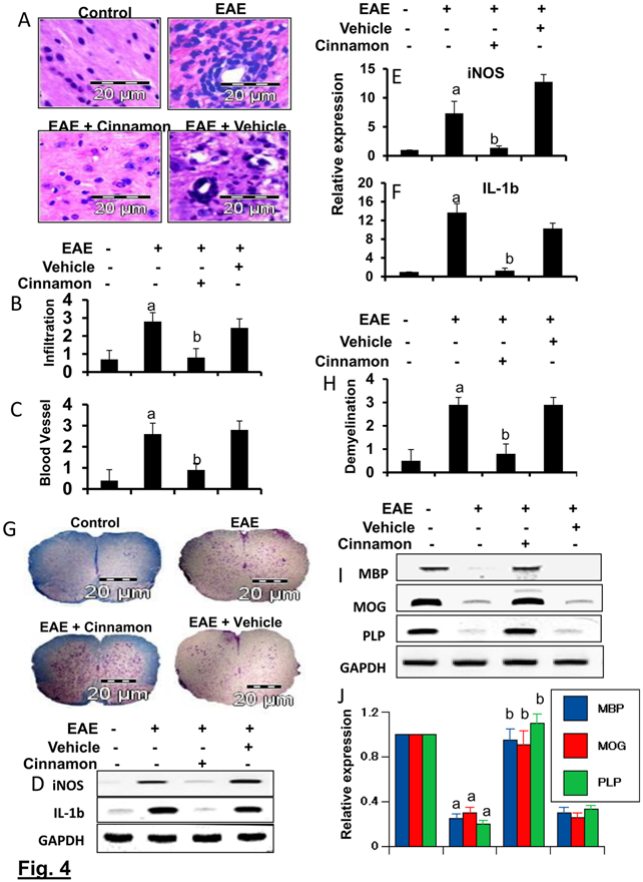
Oral administration of ground Cinnamon suppresses the infiltration of mononuclear cells and inhibits demyelination in the spinal cord of EAE in female PLP-TCR Tg mice. (A) Spinal cord sections of control, EAE (14 dpi) and either cinnamon- or vehicle-treated EAE mice (receiving cinnamon/vehicle from 8 dpi) were stained with H&E. Digital images were collected under a bright-field setting using a ×40 objective. Infiltration (B) and cuffed vessel (C) were represented quantitatively by using a scale as described by us. Data are expressed as mean ± SEM of five different mice. *^a^* *p*< 0.001 vs normal; ^b^p<0.001 vs EAE. Spinal cord of normal, EAE, and either cinnamon- or vehicle-treated EAE mice were analyzed for iNOS and IL-1β mRNAs by semi-quantitative RT-PCR (D) and quantitative real-time PCR (E for iNOS, & F for IL-1β). Data are expressed as the mean ± SEM of five different mice per group. *^a^* *p*< 0.001 vs normal, ^b^p<0.001 vs EAE. Spinal cord sections were stained with Luxol fast blue. Digital images were collected under bright field setting using a 40X objective (G). Demyelination was represented quantitatively by using a scale as described by us (H). Data are expressed as the mean ± SEM of five different mice per group; *^a^* *p*<0.001 vs normal, ^b^p<0.001 vs EAE. Spinal cord samples were analyzed for MBP, MOG, and PLP mRNAs by semi-quantitative RT-PCR (I) and real-time PCR (J). Data are expressed as the mean ± SEM of five different mice per group; *^a^* *p*<0.001 vs normal, ^b^p<0.001 vs EAE.

EAE as well as MS is caused by infiltration of autoreactive T cells and associated mononuclear cells, like macrophages, into the CNS, followed by broad-spectrum inflammatory events [9, 21]. We examined whether cinnamon attenuated infiltration and inflammation in RR-EAE in PLP-TCR transgenic mice. Mice receiving cinnamon from 8 dpi (onset of the acute phase) were sacrificed on 16 dpi. H & E staining showed widespread infiltration of inflammatory cells into the spinal cord (Fig. 4A) of RR-EAE mice. On the other hand, cinnamon treatment markedly inhibited the infiltration of inflammatory cells into the spinal cord of RR-EAE mice (Fig. 4A). In contrast, vehicle was unable to inhibit the infiltration of inflammatory cells (Fig. 4A). Quantitation of the relative level of inflammatory cells showed that cinnamon, but not vehicle, dramatically reduced infiltration (Fig. 4B) and the appearance of cuffed vessels (Fig. 4C) in spinal cord of RR-EAE mice.

Since infiltration was inhibited, we next examined whether cinnamon was capable of inhibiting the expression of proinflammatory molecules in the spinal cord of RR-EAE mice. Marked expression of pro-inflammatory molecules like iNOS and IL-1β was observed in the spinal cord of untreated RR-EAE mice compared to control mice (Fig. 4D-F). However, cinnamon treatment dramatically reduced the expression of these pro-inflammatory molecules in the spinal cord of RR-EAE mice (Fig. 4D-F).

It is believed that infiltration of blood mononuclear cells and associated neuroinflammation plays an important role in CNS demyelination observed in MS patients and EAE animals [9, 22, 23]. Therefore, we examined whether cinnamon protected RR-EAE mice from demyelination. We stained spinal cord sections by luxol fast blue (LFB) for myelin and observed widespread demyelination zones in the white matter (Fig. 4G-H). However, cinnamon treatment remarkably restored myelin level in the spinal cord of RR-EAE mice (Fig. 4G-H). In contrast, vehicle was unable to restore myelin level in spinal cord of RR-EAE mice (Fig. 4G-H). To confirm this finding, we monitored the expression of three myelin genes, MBP, MOG and PLP, and observed a marked loss of mRNA expression of these genes in the spinal cord of untreated RR-EAE mice compared to control mice (Fig. 4I-J). A significant restoration of myelin gene mRNA expression was observed in RR-EAE mice that were treated with cinnamon, but not in mice treated with vehicle (Fig. 4I-J).

**Figure 5:**
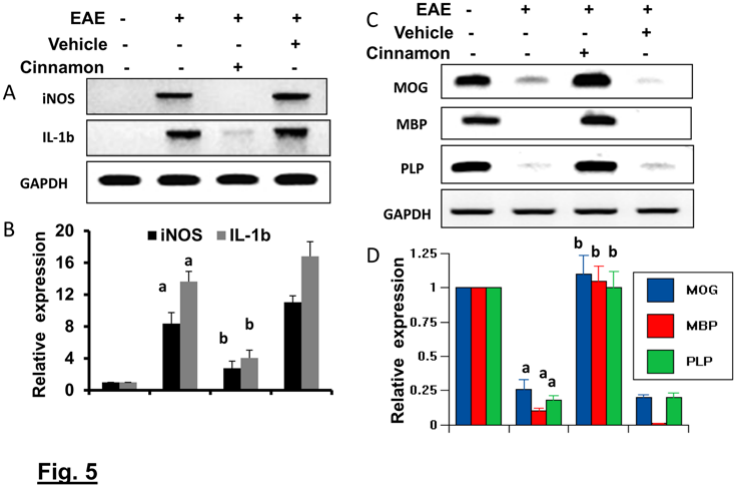
Oral administration of ground cinnamon inhibits the expression of proinflammatory molecules and upregulates the expression of myelin genes in the spinal cord of chronic EAE in C57/BL6 mice. C57BL/6 mice were immunized with 100 μg of MOG35–55, and from 8 dpi, mice were treated with either cinnamon or vehicle. On 20 dpi, spinal cord of normal, EAE, and either cinnamon- or vehicle-treated EAE mice were analyzed for mRNA expression of proinflammatory molecules ( iNOS and IL-1β) (A & B) and myelin-specific molecules (MOG, MBP and PLP) (C & D) by semi-quantitative RT-PCR (A & C) and quantitative real-time PCR (B & D). Data are expressed as the mean ± SEM of five different mice per group. *^a^* *p*< 0.001 vs normal; ^b^p<0.001 vs EAE.

Next, we investigated whether cinnamon was also able to reduce inflammation and demyelination in mice with chronic EAE (Ch-EAE). Similar to that found in RR-EAE mice, the mRNA expression of proinflammatory molecules (iNOS and IL-1β) increased (Fig. 5A-B), while the mRNA expression of myelin-specific genes (MOG, MBP and PLP) decreased in the spinal cord of mice with Ch-EAE (Fig. 5C-D). However, cinnamon treatment reduced the expression of proinflammatory molecules (Fig. 5A-B) and increased the level of myelin-specific molecules (Fig. 5C-D) in the spinal cord of Ch-EAE mice. These results demonstrate that cinnamon treatment inhibits infiltration of mononuclear cells, inflammation, and demyelination in the spinal cord of EAE mice.

### Enrichment of the Treg population by cinnamon treatment

**Figure 6:**
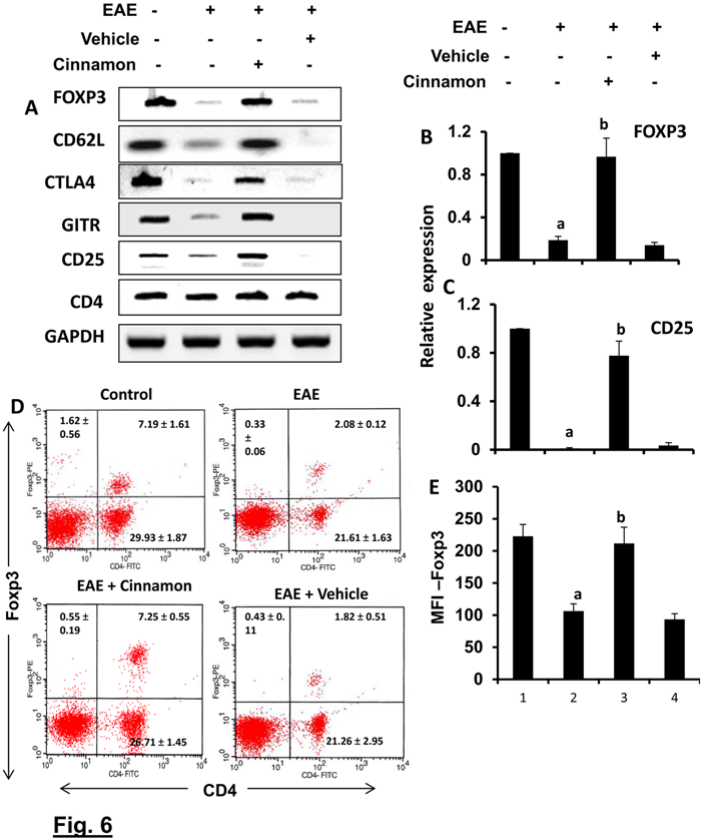
Oral administration of ground cinnamon enriches regulatory T cells (Tregs) *in vivo* in EAE in PLP-TCR Tg mice. Spleens of control, EAE (14 dpi) and either cinnamon- or vehicle-treated EAE mice (14 dpi receiving cinnamon/vehicle from 8 dpi) were analyzed for the mRNA expression of Foxp3, CD62L, CTLA4, GITR, CD25, and CD4 by semi-quantitative RT-PCR (A) and real-time PCR (Foxp3, B; and CD25, C). Data are expressed as the mean ± SEM of 5 mice per group. *^a^* *p*< 0.001 vs normal; ^b^p<0.001 vs EAE. LNC isolated from different groups of mice were analyzed by FACS for Foxp3 and CD4 (D). The MFI of Foxp3 in CD4+ population was calculated by using the CellQuest software (E). Data are mean ± SEM of 5 mice per group. *^a^* *p*<0.001 vs normal; ^b^p<0.001 vs EAE.

Because Tregs are most important immunomodulatory subtype of T lymphocytes, in order to understand immunomodulatory effect of cinnamon, at first, we examined the effect of cinnamon treatment on the status of Tregs *in vivo* in EAE mice. EAE mice receiving cinnamon or vehicle from 8 dpi were sacrificed on 16 dpi followed by analysis of Tregs in spleen and splenocytes. A major population of Tregs is characterized by a transcription factor FoxP3. During autoimmune insults, Tregs become both numerically and functionally defective. Therefore, as expected, we observed marked loss of Foxp3 in the spleen of EAE mice as compared to control mice (Fig. 6A). Because Foxp3+ T cells usually express CD25, CTLA4, CD62L, and GITR, we also analyzed the mRNA expression of these molecules. Similar to Foxp3, the mRNA expression of CD25, CD62L, CTLA4, and GITR also decreased in the spleen of EAE mice as compared to normal mice (Fig. 6A). However, treatment of EAE mice with cinnamon, but not vehicle, led to the protection of Foxp3, CD25, CD62L, CTLA4, and GITR as evident from our semi-quantitative RT-PCR (Fig. 6A). RT-PCR results for Foxp3 and CD25 mRNAs were also confirmed by real-time PCR (Fig. 6B-C). On the other hand, either the induction of EAE or cinnamon treatment had no effect on the mRNA expression of CD4 (Fig. 6A), suggesting that these results are not due to any alteration in CD4+ T cells.

To confirm these results further, we performed FACS analysis. As expected, there was a significant reduction in Foxp3+CD4+ population of T cells in EAE splenocytes as evident from FACS dot plot (Fig. 6D) and mean fluorescence intensity (MFI) (Fig. 6E). However, treatment of EAE mice with cinnamon, but not vehicle, led to the increase in Foxp3+CD4+ population in splenocytes (Fig. 6E). MFI calculation in Fig. 6E also shows that cinnamon treatment resulted in significant increase in Foxp3.

**Figure 7:**
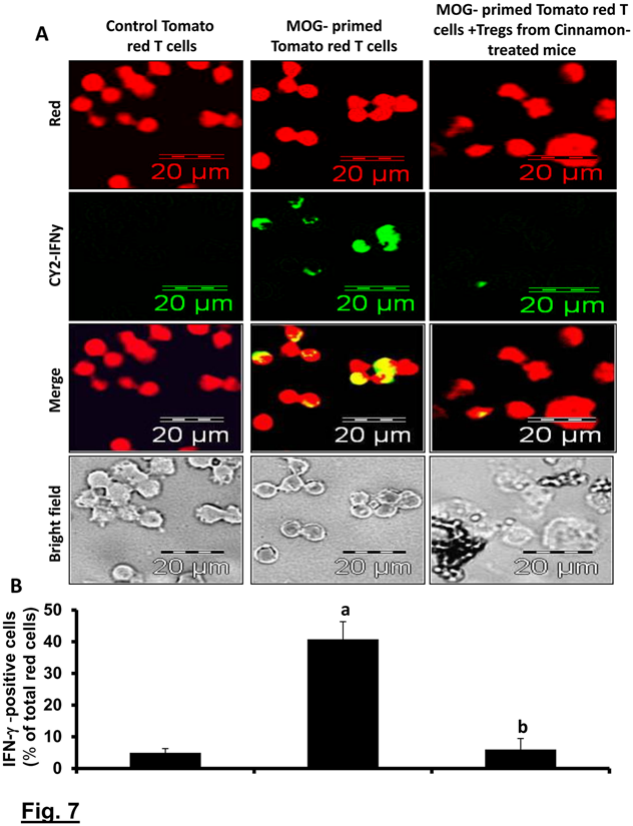
Suppressive activity of Tregs isolated from cinnamon-treated EAE mice. Tomato red transgenic mice were immunized with MOG (100 µg/mouse) and 12 d after immunization; splenocytes were isolated and re-primed with MOG (10 µg/ml) for 2 d. Similarly, C57BL/6 mice were also immunized with MOG and from 8 dpi, mice were treated with ground cinnamon via gavage. On 16 dpi, splenocytes were isolated and re-primed with MOG for 2d followed by purification of CD4+CD25+ Tregs. Then these Tregs were added to MOG-primed splenocytes isolated from tomato red transgenic mice at a ratio of 2:1 of tomato red T cell:Tregs. After 24 h, non-adherent cells were immunostained for IFN-γ (green). Cells from tomato red transgenic mice exhibited red color (A). IFN-γ expressing red cells were counted and expressed as percent of total red cells (B). Data are mean ± SEM of 20 different images. *^a^* *p*< 0.001 vs control tomato red T cells, ^b^p<0.001 vs MOG-primed tomato red T cells.

To understand that cinnamon-induced Tregs in EAE mice were functionally active, we examined the suppressive activity of these Tregs. As evident from Fig. 7A-B, IFN-γ expression was very low in normal tomato red T cells and MOG priming significantly increased the expression of IFN-γ in these T cells. However, cinnamon-induced MOG-primed Tregs of B6.129 mice markedly suppressed the expression of IFN-γ in MOG-primed tomato red T cells (Fig. 7A-B). This result was specific as MOG-primed non-Tregs increased the expression of IFN-γ in tomato red T cells (data not shown). These results demonstrate that cinnamon-induced Tregs in EAE mice are functionally active.

### How does cinnamon enrich Tregs?

**Figure 8:**
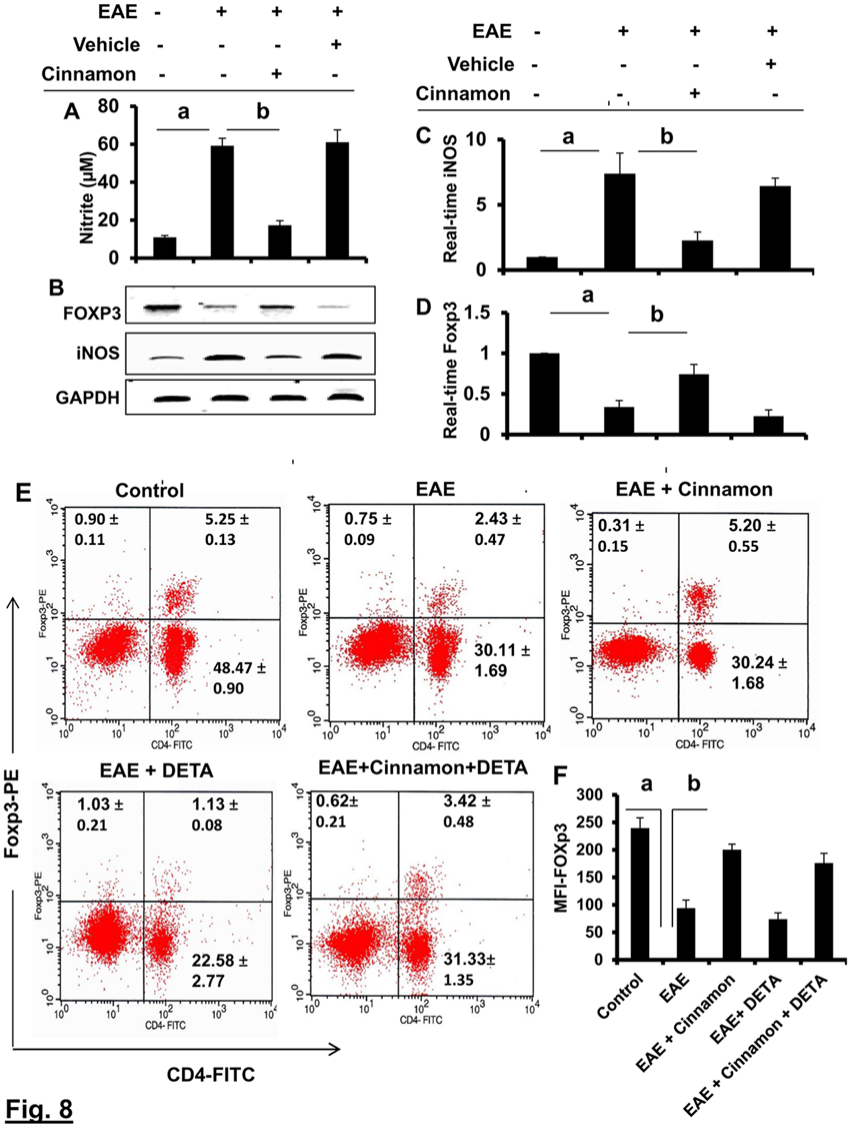
Cinnamon treatment enriches Tregs in EAE in PLP-TCR Tg mice via suppressing NO production. Splenocytes isolated from control, EAE (14 dpi) and either cinnamon- or vehicle-treated EAE mice (14 dpi receiving cinnamon/vehicle from 8 dpi) were re-primed with PLP139–151 for 24 h followed by monitoring the level of nitrite in supernatants (A) and the mRNA expression of iNOS and Foxp3 by semi-quantitative RT-PCR (B) and real-time PCR (C, iNOS; D, Foxp3). Data are expressed as the mean ± SEM of 3 mice per group. *^a^* *p*< 0.001 vs control; ^b^p<0.001 vs EAE. Splenocytes isolated from cinnamon-treated EAE mice (14 dpi receiving cinnamon/vehicle from 8 dpi) were re-primed with PLP139–151 for 24 h in the presence or absence of DETA-NONOate (a NO donor) followed by FACS for Foxp3 and CD4 (E). The MFI of Foxp3 in CD4+ population was calculated by using the CellQuest software (F). Data are mean ± SEM of 3 mice per group. *^a^* *p*<0.001 vs normal; ^b^p<0.001 vs EAE; ^c^p<0.001 vs (EAE+cinnamon).

Recently, we have reported that NO is a critical regulator of Tregs [17]. Therefore, we were prompted to investigate whether cinnamon treatment increased the number of Tregs via decreasing NO. At first, we examined whether cinnamon treatment would inhibit the expression of iNOS and the production of NO in the spleen of EAE mice. As expected, splenocytes isolated from EAE mice produced 5-fold more NO (measured as nitrite) as compared to that isolated from control mice (Fig. 8A). However, treatment of EAE mice with cinnamon, but not vehicle, led to the suppression of NO production in splenocytes (Fig. 8A). To understand the mechanism further, we investigated the effect of cinnamon on mRNA level of iNOS. It is evident from semi-quantitative RT-PCR (Fig. 8B) and real-time PCR (Fig. 8C) analyses that treatment of EAE mice with cinnamon, but not vehicle, led to the inhibition of iNOS mRNA expression in splenocytes. These results suggest that cinnamon is capable of suppressing the expression of iNOS in EAE mice. Next, we monitored the level of Foxp3 mRNA. In contrast to iNOS, induction of EAE decreased the mRNA expression of Foxp3, whereas cinnamon blocked the loss of Foxp3 in splenocytes (Fig. 8B for RT-PCR and Fig. 8D for real-time PCR).

Next, to directly test a role of NO in cinnamon-mediated modulation of Foxp3, we added DETA-NONOate (an NO donor) to splenocytes isolated from cinnamon-treated EAE mice. It is evident from RT-PCR (Fig. 8B), real-time PCR (Fig. 8D), FACS dot plot (Fig. 8E), and MFI analysis (Fig. 8F) that cinnamon treatment of EAE mice resulted to an increase in the level of Foxp3 in splenocytes. However, this increase and/or protection of Foxp3 mRNA and protein was completely abrogated by DETA-NONOate (Fig. 8B, D-F), indicating an important role of NO in cinnamon-mediated upregulation of Foxp3 and enrichment of Tregs.

### Suppression of the Th17 response by cinnamon treatment

**Figure 9:**
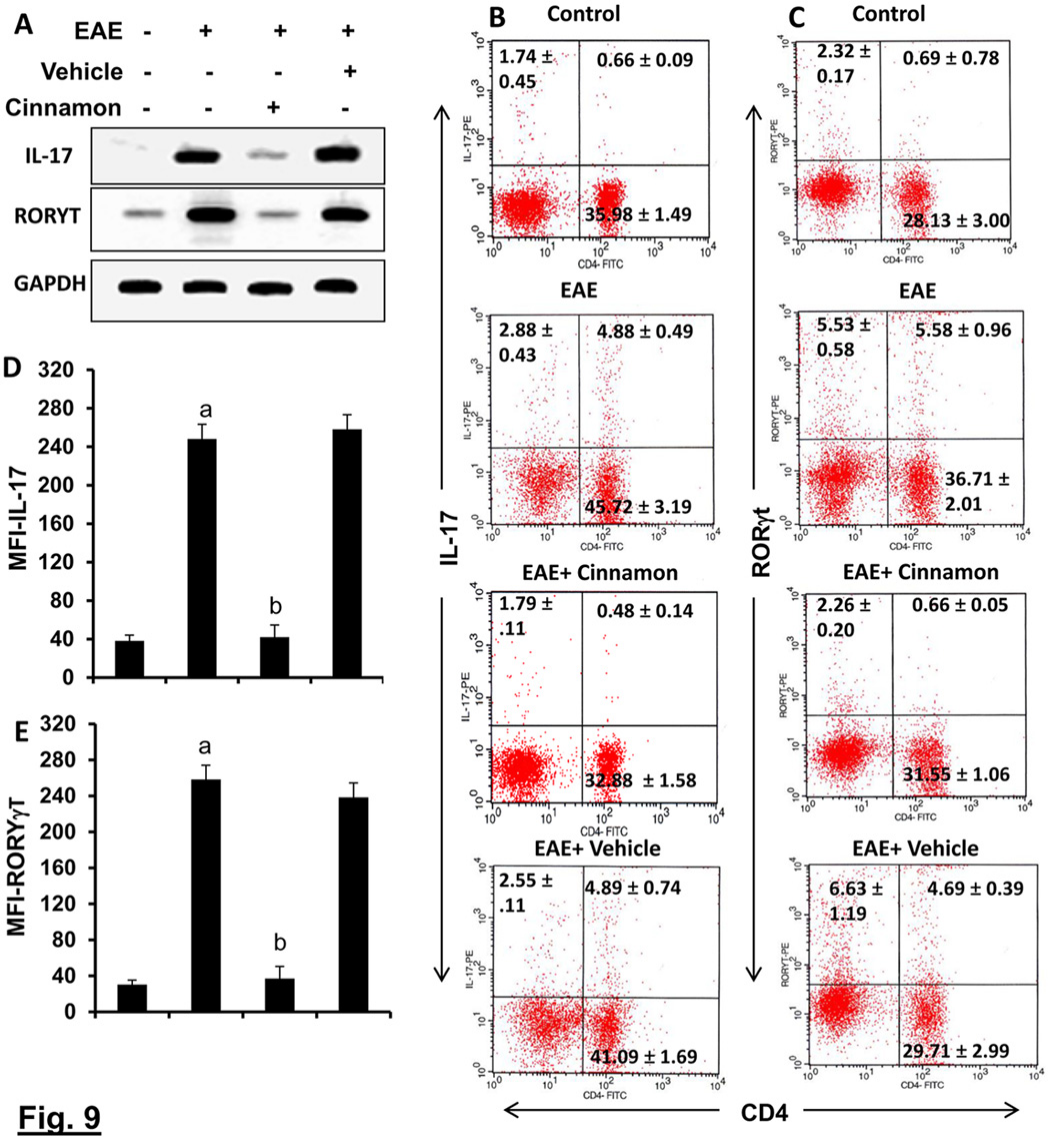
Oral administration of ground cinnamon suppresses autoimmune Th17 cells *in vivo* in EAE in PLP-TCR Tg mice. Spleens of control, EAE (14 dpi) and either cinnamon- or vehicle-treated EAE mice (14 dpi receiving cinnamon/vehicle from 8 dpi) were analyzed for the mRNA expression of Th17-specific factors (RORγt and IL-17) (A). Splenocytes isolated from different groups of mice were analyzed by FACS for RORγt & CD4 (B) and IL-17 & CD4 (C). The MFI of RORγT (D) and IL-17 (E) in CD4+ population were calculated by using the CellQuest software. Data are mean ± SEM of 3 mice per group. *^a^* *p*< 0.001 vs normal; ^b^p<0.001 vs EAE.

After the discovery of IL-23, Th17 cells are considered to play a more active role than Th1 cells in the disease process of EAE and MS [24]. It has been found that there is an inverse relationship between Th17 cells and Tregs [25]. Because cinnamon treatment enriched Tregs, we examined whether cinnamon was capable of regulating Th17 cells in EAE mice. While induction of EAE increased the expression of IL-17 mRNA (Fig. 9A) and the level of CD4+IL-17+ T cells in splenocytes (Fig. 9B), cinnamon markedly suppressed EAE-induced upregulation of IL-17 mRNA (Fig. 9A) as well as the CD4+IL-17+ T cell population (Fig. 9B). On the other hand, vehicle treatment had no such suppressive effects on CD4+IL-17+ T cell (Fig. 9B). MFI analysis of IL-17 (Fig. 9D) within the CD4+ population also supported this finding. Th17 cells are also characterized by a transcription factor called RORγT [26]. To confirm the regulation of Th17 cells by cinnamon treatment, we also monitored RORγT. Consistent with the upregulation of IL-17 mRNA and CD4+IL-17+ T cells, induction of EAE increased the mRNA expression of RORγT (Fig. 9A) and the level of CD4+RORγT+ T cells in splenocytes (Fig. 9C) and treatment of EAE mice with cinnamon, but not vehicle, resulted in suppression of RORγT mRNA (Fig. 9A) and CD4+RORγT+ T cells (Fig. 9C). This is also corroborated by MFI analysis of RORγT within the CD4+ population (Fig. 9E). These results clearly show that cinnamon is capable of suppressing Th17 cells.

### Switching of Th1 to Th2 in response to cinnamon treatment

**Figure 10:**
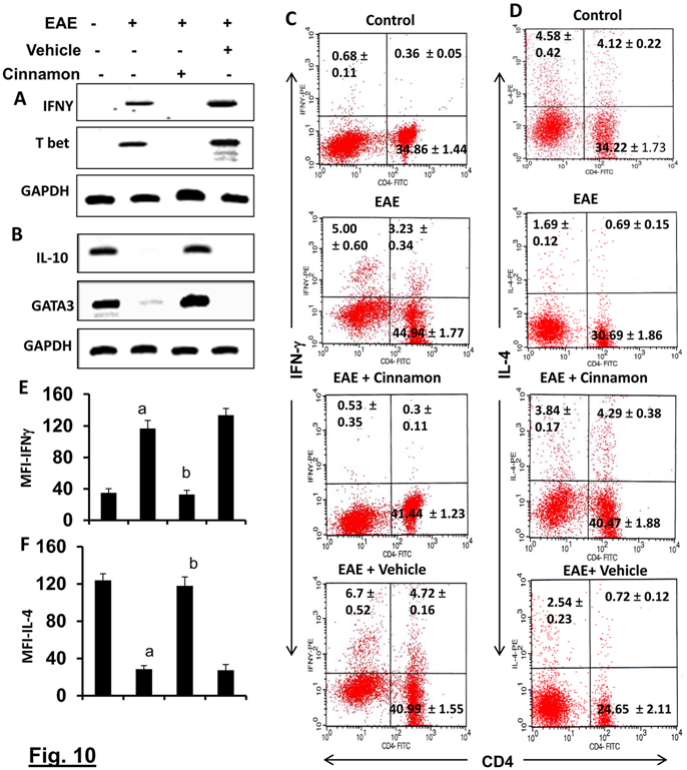
Oral administration of ground cinnamon switches Th1 to Th2 *in vivo* in EAE in PLP-TCR Tg mice. Spleens of control, EAE (14 dpi) and either cinnamon- or vehicle-treated EAE mice (14 dpi receiving cinnamon/vehicle from 8 dpi) were analyzed for the mRNA expression of Th1-specific factors (Tbet and IFN-γ) (A) and Th2-specific factors (GATA3 and IL-10) (B) by semi-quantitative RT-PCR. LNC isolated from different groups of mice were analyzed by FACS for IFN-γ & CD4 (C) and IL-4 & CD4 (D). The MFI of IFN-γ (E) and IL-4 (F) in CD4+ population were calculated by using the CellQuest software. Data are mean ± SEM of 3 mice per group. *^a^* *p*< 0.001 vs normal; ^b^p<0.001 vs EAE.

Similar to Th17 cells, Th1 cells are also autoimmune inflammatory and switching of the Th1 to a Th2 phenotype is one of the ways to ameliorate the disease [10, 24, 27]. Because cinnamon increased Tregs, which are known to suppress Th1 cells via releasing TGF-β and IL-10, we examined whether cinnamon treatment was capable of suppressing the autoimmune Th1 response in EAE mice. While T-bet-dependent IFN-γ production is a characteristic of Th1 cells, Th2 cells display GATA3-dependent IL-10 and IL-4 release [9, 28, 29]. As expected, induction of EAE increased the mRNA expression of IFN-γ and Tbet (Fig. 10A) and the level of CD4+IFN-γ+ T cells in splenocytes (Fig. 10C). Treatment of EAE mice with cinnamon, but not vehicle, led to marked suppression of EAE-induced upregulation of IFN-γ and Tbet mRNAs (Fig. 10A) as well as the CD4+IFN-γ+ T cell population (Fig. 10C). MFI analysis of IFN-γ (Fig. 10E) within the CD4+ population also supported this finding. Next, we analyzed the Th2 responses by monitoring Th2 cytokines and Th2-specific transcription factor GATA3. The induction of EAE suppressed the mRNA expression of IL-10 and GATA3 (Fig. 10B) and the level of CD4+IL-4+ T cells (Fig. 10D) in splenocytes. However, cinnamon treatment markedly increased the mRNA expression of IL-10 and GATA3 and the level of CD4+IL-4+ T cells in splenocytes (Fig. 10B&D). MFI analysis of IL-4 (Fig. 10F) within the CD4+ population also supported this finding. This effect was specific as vehicle treatment had no effect. Together, these results suggest that cinnamon treatment was capable of suppressing the Th1 response, while augmenting the Th2 response in EAE mice.

### Cinnamon suppresses EAE in mice via Tregs

**Figure 11:**
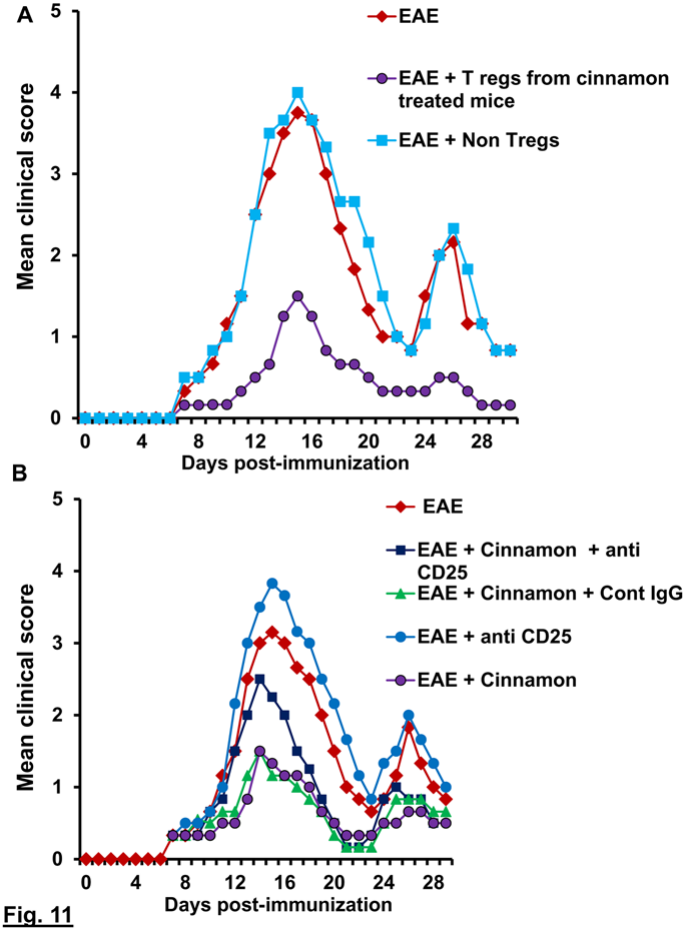
Oral administration of cinnamon protects EAE in PLP-TCR Tg mice via Tregs. Female PLP-TCR Tg mice were immunized with 10 μg of PLP139–151, and from 8 dpi, mice were treated with cinnamon (100mg/kg of body weight/d) via gavage. After 8 d of treatment, splenocytes were isolated followed by purification of CD4+CD25+ Tregs. In a parallel experiment, EAE was induced in female PLP-TCR Tg mice and from 4 dpi, EAE mice were treated (i.p.) once with cinnamon-induced Tregs (1×10^6^ Tregs/mouse). Purified CD4+CD25- non-Tregs were also used for comparison. Six recipient mice (n = 6) were included in each group. Mice were examined for clinical symptoms daily until 30 dpi (A). EAE was induced in female PLP-TCR Tg mice and from 2 dpi, mice were treated with cinnamon (100mg/kg of body weight/d) via gavage followed by one i.p. injection of anti-CD25 antibody (50 µg/mouse) on the same day. One group of mice also received same amount of control IgG. Mice (n = 6) were examined for clinical symptoms daily.

Next, in order to test the functional significance of cinnamon-mediated increase in Treg activity, we examined whether cinnamon protected mice from clinical symptoms of EAE via Tregs. At first, we checked whether cinnamon-induced Tregs from donor mice were capable of suppressing the adoptive transfer of EAE in female SJL/J mice. A single injection of cinnamon-induced Tregs on 4 dpt markedly suppressed the clinical symptoms of EAE in recipient mice in acute as well as relapsing phases of the disease (Fig. 11A). This result was specific, as CD4+CD25- non-Tregs remained unable to inhibit the disease process of RR-EAE (Fig. 11A). These results suggest cinnamon-induced Tregs are capable of suppressing EAE.

Next, we examined whether cinnamon treatment also protected EAE via Tregs. Therefore, during cinnamon treatment, the function of Tregs was blocked *in vivo* in EAE mice by anti-CD25 antibody. As evident from Fig. 11B, cinnamon treatment ameliorated clinical symptoms of RR-EAE. However, functional blocking anti-CD25 antibody almost completely abrogated the cinnamon-mediated protective effect on EAE mice (Fig. 11B). This result was specific as control IgG had no such effect (Fig. 11B). Together, these results delineate an important role of Tregs in cinnamon-mediated protection of EAE.

## Discussion

MS is the most common human demyelinating disease of the CNS and despite intense investigations, no effective therapy is available for this disease. Tysabri and different forms of interferon-β (IFN-β) are currently used to treat MS. However, reduced effectiveness and severe toxic effects over chronic use, as well as treatment costs, often limit these available therapies. For example, IFN-β has a number of side effects including flu-like symptoms, menstrual disorders in women, decrease in neutrophil and white blood cell count, increase in AST and ALT levels, and development of neutralizing antibodies to IFN-β [9, 30, 31]. Similarly, treatment with Tysabri can cause lung infection, breathing problems, chest pain, wheezing, urinary tract infection, vaginitis, nausea, vomiting, and liver damage. Tysabri also increases the chance of getting a severe brain infection, leading to progressive multifocal encephalopathy, which may cause disability and death. Therefore, it is necessary to identify safe, effective and economical therapeutic option for MS.

Cinnamon, the brown bark of cinnamon tree, is a commonly used spice and flavoring material for deserts, candies, chocolate, etc. It has a long history as a medicine as well. Medieval physicians used cinnamon in medicines to treat a variety of disorders including arthritis, coughing, hoarseness, sore throats, etc. In addition to containing manganese, dietary fiber, iron, and calcium, cinnamon contains a major compound, cinnamaldehyde, which is converted into cinnamic acid by oxidation. In the liver, this cinnamic acid is β-oxidized to benzoate [32] that exists as sodium salt (NaB) or benzoyl-CoA. It has been reported that minor amount of NaB is also excreted in the urine of human [33, 34]. NaB is of medical importance as it is a component of Ucephan, a FDA-approved drug used in the treatment for hepatic metabolic defects associated with hyperammonemia such as urea cycle disorder in children [35, 36]. It is also widely used as a preservative in broad range of foods and cosmetic products [37]. Earlier we have demonstrated that NaB modifies T cells at multiple steps and protects experimental allergic encephalomyelitis, an animal model of MS [13]. Here we provide the first evidence that oral feeding of cinnamon powder is capable of suppressing the disease process of EAE in mice. Our conclusion is based on the following observations. PLP immunization remained unable to induce clinical symptoms of EAE in female PLP-TCR transgenic mice receiving cinnamon orally. In contrast, PLP priming induced EAE in PLP-TCR mice receiving methyl cellulose (vehicle). From a therapeutic point of view, it is important to test whether a drug candidate is efficacious when administered after the onset of disease symptoms. Oral cinnamon powder fulfilled this requirement and inhibited the progression of RR-EAE when administered either early or at a late stage of the disease progression. Therapeutic treatment of EAE animals with cinnamon was also capable of inhibiting the invasion of mononuclear cells into the spinal cord, as well as the expression of inflammatory molecules (iNOS and IL-1β), and restored myelination and the expression of myelin genes within the CNS. We did not notice any side effect (e.g. hair loss, weight loss, untoward infection etc.) in any of the mice used during cinnamon treatment. These results suggest that cinnamon may be considered to mitigate the disease process in MS patients.

It has been shown that there is a significant decrease in the number of CD4^+^Foxp3^+^ T cells as well as the expression level of Foxp3 in relapsing-remitting MS and other lymphoproliferative autoimmune disorders [6, 8, 38]. Hence, the upregulation of Foxp3+ Tregs might be useful for suppressing the activation of autoimmune Th1 and Th17 cells and controlling autoimmune disorders. Here, we provide the first evidence that oral feeding of cinnamon powder is capable of enriching Foxp3^+^ Tregs *in vivo* in mice. As reported earlier, induction of EAE reduced the expression of Foxp3 in spleen. However, cinnamon treatment markedly inhibited the loss of Foxp3 in the spleen of EAE mice. This was also verified by dual-label (CD4 and Foxp3) FACS analysis of LNC. Furthermore, Foxp3+ Tregs are also characterized by CD25, CD62L, CTLA4, GITR etc. Accordingly, we have found loss of CD25, CD62L, CTLA4, and GITR in the spleen of EAE mice as compared to normal mice. Again, cinnamon treatment inhibited the loss of CD25, CD62L, CTLA4, and GITR in the spleen of EAE mice. These results were specific as neither EAE induction nor cinnamon treatment had any effect on CD4. The unperturbed level of CD4 probably implies that either suppression of Foxp3 and other Treg markers in EAE or upregulation of Foxp3 and Treg markers by cinnamon treatment is not due to any reduction of CD4^+^ cells. Finally, Tregs are also known as suppressor T cells as they suppress immune responses of other cells. Accordingly, suppression of IFN-γ expression in MOG-primed tomato red T cells by cinnamon-induced Tregs suggests that cinnamon-induced Tregs are functionally active.

How does cinnamon induce Tregs? Recently, we have demonstrated that NO negatively regulates Foxp3 and Tregs [17]. While blocking NO either by inhibiting iNOS or direct scavenging of NO or by pharmacological drugs restores the expression of Foxp3 in MBP-primed T cells, NO donors decrease Foxp3 [17]. Therefore, we hypothesized that cinnamon treatment could enrich Tregs via regulating NO production. Suppression of iNOS expression and NO production in splenocytes of cinnamon-treated EAE mice as compared to vehicle treatment and abrogation of cinnamon-mediated protection of Foxp3 by NO donor suggest that cinnamon treatment protects Tregs via suppression of NO production. Recently we have shown that oral administration of cinnamon (*Cinnamonum verum*) produces sodium benzoate (NaB) in serum and brain of mice [15]. We have also reported that NaB attenuates the production of NO [39] and that NaB restores Foxp3 in MBP-primed splenocytes via suppression of NO [17]. Therefore, it is likely that after oral administration, cinnamon is metabolized to sodium benzoate, which then suppresses the production of NO in spleen to protect Tregs in EAE mice. This suggestion is supported by the fact that NaB also enriches Tregs and protect mice from EAE [13].

Being the master regulator, Tregs are known to maintain immune homeostasis. While in one hand, Tregs suppress the proliferation of autoimmune Th1 cells by secreting TGF-β and IL-10, on the other, Tregs release IL-35 to control the proliferation of autoimmune Th17 cells [1, 3, 38, 40, 41]. Accordingly, oral cinnamon treatment suppressed both Th1 and Th17 immune responses in PLP-TCR mice. Theoretically, Tregs should also suppress Th2 via releasing TGF-β and IL-35 [3, 41, 42]. However, in our study, cinnamon treatment suppressed autoimmune Th1 and Th17 responses, while stimulating the anti-autoimmune Th2 response in EAE. It is also possible as Tregs may contribute to Th2 polarization. For example, McKee and Pearce [43] have demonstrated that Tregs contribute to Th2 polarization during Helminth infection by suppressing the development of Th1 response. Similarly, Kohm et al [44] have shown that supplementation of Tregs by adoptive transfer before EAE induction significantly reduces the severity of clinical disease, potentially by promoting anti-autoimmune Th2 response. Here, we also must remember that Tregs produce substantial amount of IL-10, a cytokine that is also produced by Th2 cells. Therefore, whether the stimulation of Th2 response by cinnamon treatment is a direct effect of cinnamon metabolites, an indirect effect via enrichment of Tregs, or both, needs further study.

Because Tregs have been implicated in the pathogenesis of autoimmune diseases, we examined the effect of cinnamon-induced Tregs in EAE mice and, in a different experiment, used an anti-CD25 antibody in cinnamon-treated EAE mice. Suppression of EAE by cinnamon-induced Tregs and abrogation of cinnamon-mediated protection of EAE by anti-CD25 antibody clearly suggest that cinnamon-induced Tregs are capable of ameliorating EAE and that the effect of cinnamon treatment is Treg-dependent.

In summary, we have demonstrated that oral administration of cinnamon powder upregulates anti-autoimmune Treg/Th2 cells, down-regulates autoimmune Th17/Th1 cells and blocks the disease process of EAE when administered either prophylactially or therapeutcially. These results highlight a novel immunomodulatory role of cinnamon and suggest that this widely-used spice may be explored for therapeutic intervention in MS.
